# IL-33 blockade suppresses tumor growth of human lung cancer through direct and indirect pathways in a preclinical model

**DOI:** 10.18632/oncotarget.19786

**Published:** 2017-08-02

**Authors:** Kailing Wang, Shan Shan, Zongjun Yang, Xia Gu, Yuanyuan Wang, Chunhong Wang, Tao Ren

**Affiliations:** ^1^ Department of Respiratory Medicine, East Hospital, Tongji University School of Medicine, Shanghai 200120, China; ^2^ Department of Clinical Laboratory, Qingdao Women & Children Hospital, Qingdao 266034, China; ^3^ Department of Respiratory Medicine, Shanghai Jiao Tong University Affiliated Sixth People’s Hospital, Shanghai 200233, China

**Keywords:** lung cancer, IL-33, tumor-associated macrophage, regulatory T cell

## Abstract

Non-small-cell lung cancer (NSCLC) is the most common type in lung cancer, a leading cause of cancer-related death worldwide. Our previous study unraveled a pro-cancer function of IL-33 in fueling outgrowth and metastasis of human NSCLC cells. Herein, we determined that interfere with IL-33 activity was an effective strategy for limiting NSCLC tumor growth using a preclinical model with human NSCLC xenografts. IL-33 blockade efficiently inhibited tumor growth of NSCLC xenografts in immune-deficient mice. Mechanistically, IL-33 blockade suppressed outgrowth capacity of human NSCLC cells. Meanwhile, IL-33 blockade abrogated polarization of M2 tumor-associated macrophages (TAMs) and reduced accumulation of regulatory T cells (Tregs) in tumor microenvironments, shaping functional immune surveillance. In NSCLC patients, IL-33 expressions were positively correlated with Ki-67 proliferation index and expressions of M2 TAM- and Teg-related genes. These findings identify IL-33 as a dual-functional factor in NSCLC pathogenesis and suggest IL-33 blockade as a promising therapeutic for NSCLC patients.

## INTRODUCTION

Lung cancer is the leading cause of cancer-related death worldwide [1]. Among patients with lung cancer, non-small cell lung cancer (NSCLC) accounts for about 85% of all clinical cases [1, 2]. Besides the traditional therapeutics including surgery and chemotherapy, targeted therapy is currently considered as an optimal treatment for NSCLC patients [3, 4]. The application of targeted drugs is based on accurate NSCLC genetic mutations, which are crucial for tumor initiation and progression [3–5]. However, the majority of NSCLC patients are wild type and not suitable be treated by targeted drugs [6]. Resistance to targeted drugs seems common and inevitable, dampening the effectiveness of targeted therapy [6–8]. Currently, the prognoses of NSCLC patients are far less satisfied [9]. New therapeutic explorations are urgently needed for optimizing treatment of NSCLC patients.

Interleukin-33 (IL-33) is one new member of IL-1 superfamily and functions either as a nuclear transcriptional factor or as a released cytokine [10, 11]. IL-33 maintains barrier functions as a nuclear protein in physiological conditions and participates in pathogenic diseases as an alarming cytokine by binding to its receptor ST2 [10–12]. Recent studies identify IL-33 as an important pro-cancer factor. For instance, IL-33 is able to promote the growth and metastasis of colorectal cancer, breast cancer, gastric cancer and ovarian cancer [13–19]. In consistent, our previous study reported a crucial role of IL-33 in driving NSCLC outgrowth and metastasis through regulating membrane GLUT1 localization and aerobic glycolysis, suggesting IL-33 as a promising target for treatment of NSCLC patients [20].

Current studies investigating therapeutic efficiency of anti-cancer reagents are heavily relied on murine models with established human tumor cell lines, frequently resulting in difficulties from translating the findings into clinical application because tumor cell lines do not maintain the characteristics and heterogeneity of cancer cells from clinical patients [21, 22]. Herein, we evaluate the application of IL-33 based therapeutic in treating NSCLC patients using a pre-clinical mouse model. By engraftment of tumor tissues from NSCLC patients into immune-deficient mice and subsequent treatment of tumor-bearing mice with IL-33 blockers, we demonstrate that blockade of IL-33 is an efficient strategy to limit NSCLC growth. The mechanism involves direct effect by dampening NSCLC proliferative survival and indirect effect by inhibiting polarization of M2 macrophages and reducing accumulation of regulatory T cells in tumor microenvironments. These findings obtained from human NSCLC tumor xenograft model reflect a realistic condition *in vivo*, unraveling IL-33 blockade as a novel option for optimizing treatment of NSCLC patients.

## RESULTS

### Establishment of patient-derived NSCLC tumor xenograft model

**Figure 1:**
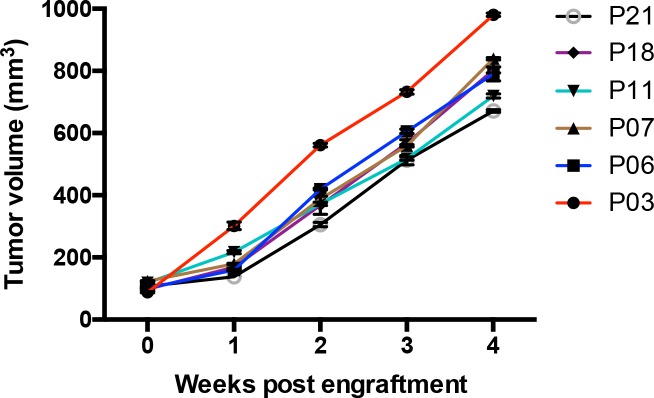
Tumor growth of human NSCLC xenografts Tumor volumes of NSCLC xenografts in the third generation were detected at the indicated time post engraftment. Each tumor was measured by two researchers. Shown are mean±SEM from 6 patients samples.

Fresh NSCLC tumor fragments from 22 patients were implanted subcutaneously into SCID mice and monitored for tumor growth. The tumor xenografts removed and passaged to nude mice were established from 6 patient-derived samples, showing stable tumor growth in nude mice in the third generation (Figure 1). These established NSCLC xenograft models were used to evaluate the efficacy of IL-33 based therapeutics.

### IL-33 blockade limits tumor growth of NSCLC tumor xenograft

**Figure 2:**
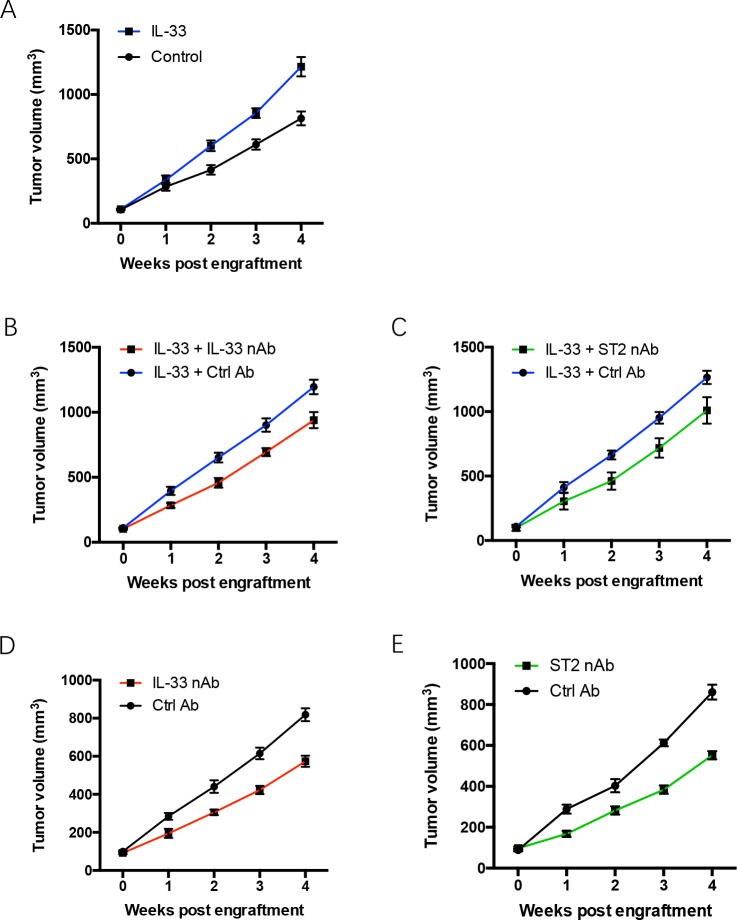
IL-33 blockade restricts tumor growth of NSCLC xenografts **(A)** NSCLC xenografts were treated with recombinant human IL-33 protein or the control and detected for tumor growth at the indicated time. **(B, C)** NSCLC xenografts were treated with recombinant human IL-33 protein plus IL-33 neutralizing antibody (B) or ST2 neutralizing antibody (C). **(D, E)** NSCLC xenografts were treated with IL-33 neutralizing antibody (D) or ST2 neutralizing antibody (E) and analyzed for tumor growth. Shown are mean±SEM from 6 independent experiments.

To determine the effect of IL-33 based therapeutics in treatment of NSCLC, nude mice bearing the fourth generation of human NSCLC tumors were treated with recombinant IL-33 protein plus IL-33 neutralizing antibody or ST2 neutralizing antibody. While tumor growth of untreated NSCLC xenografts was stable *in vivo*, it was significantly enhanced by IL-33 treatment (Figure 2A). The function of IL-33 in promoting NSCLC xenograft growth could be abrogated by IL-33 neutralizing antibody and ST2 neutralizing antibody (Figure 2B, 2C). Of note, IL-33 blockade efficiently alleviated tumor growth of untreated NSCLC xenografts (Figure 2D, 2E). These data suggest IL-33 blockade as an effective therapeutic strategy for human NSCLC.

### IL-33 blockade inhibits proliferative survival of NSCLC cells

**Figure 3:**
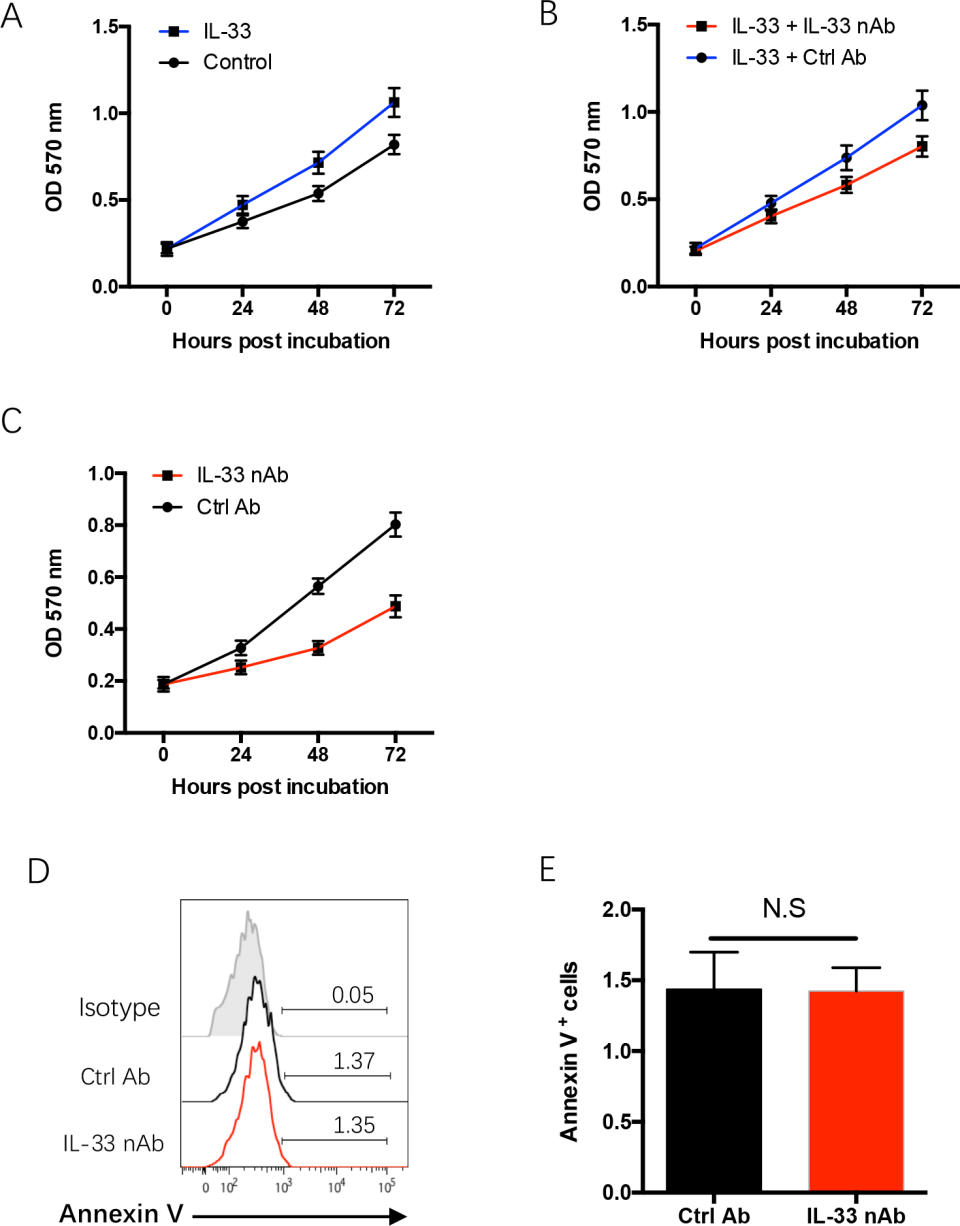
IL-33 confers NSCLC proliferative expansion **(A)** Freshly isolated NSCLC cells were treated with recombinant human IL-33 protein (20 ng/ml) or the control and analyzed for their proliferative growth with MTT assay. Shown are mean±SEM from 4 independent experiments. **(B)** Freshly isolated NSCLC cells were treated with recombinant human IL-33 protein (20 ng/ml) plus IL-33 blocking antibody (10μg/ml) and analyzed for their proliferative growth. Shown are mean±SEM from 4 independent experiments. **(C)** Freshly isolated NSCLC cells were treated with IL-33 blocking antibody (10μg/ml) or the control and analyzed for their proliferative growth. Shown are mean±SEM from 4 independent experiments. **(D, E)** Freshly isolated NSCLC cells were treated with IL-33 neutralizing antibody (10μg/ml) or the control antibody for 24h and analyzed for their apoptosis with Annexin V staining. Representative histograms and collective data (mean±SEM) from 4 independent experiments are shown.

To understand the mechanisms underlying limited NSCLC growth by IL-33 blockade, freshly isolated NSCLC cells from surgical tissues were incubated with recombinant human IL-33 protein with or without IL-33 neutralizing antibody and analyzed for their proliferative survival by MTT assay. IL-33 promoted outgrowth capacity of human NSCLC cells, which could be blocked by IL-33 neutralization (Figure 3A, 3B). Further, IL-33 neutralization itself exerted an inhibitory function in NSCLC proliferative survival (Figure 3C), suggesting that IL-33 promotes NSCLC outgrowth through an autocrine manner. These data were in line with our previous study that showed a robust expression of IL-33 and ST2 in tumor cells of NSCLC patients [20]. In contrast, we did not observe a significant effect of IL-33 blockade on NSCLC apoptosis (Figure 3D, 3E). Collectively, IL-33 blockade suppresses NSCLC progression, at least partly, through a direct inhibition on NSCLC outgrowth.

### IL-33 blockade abrogates polarization of M2 tumor-associated macrophages in tumor microenvironment

**Figure 4:**
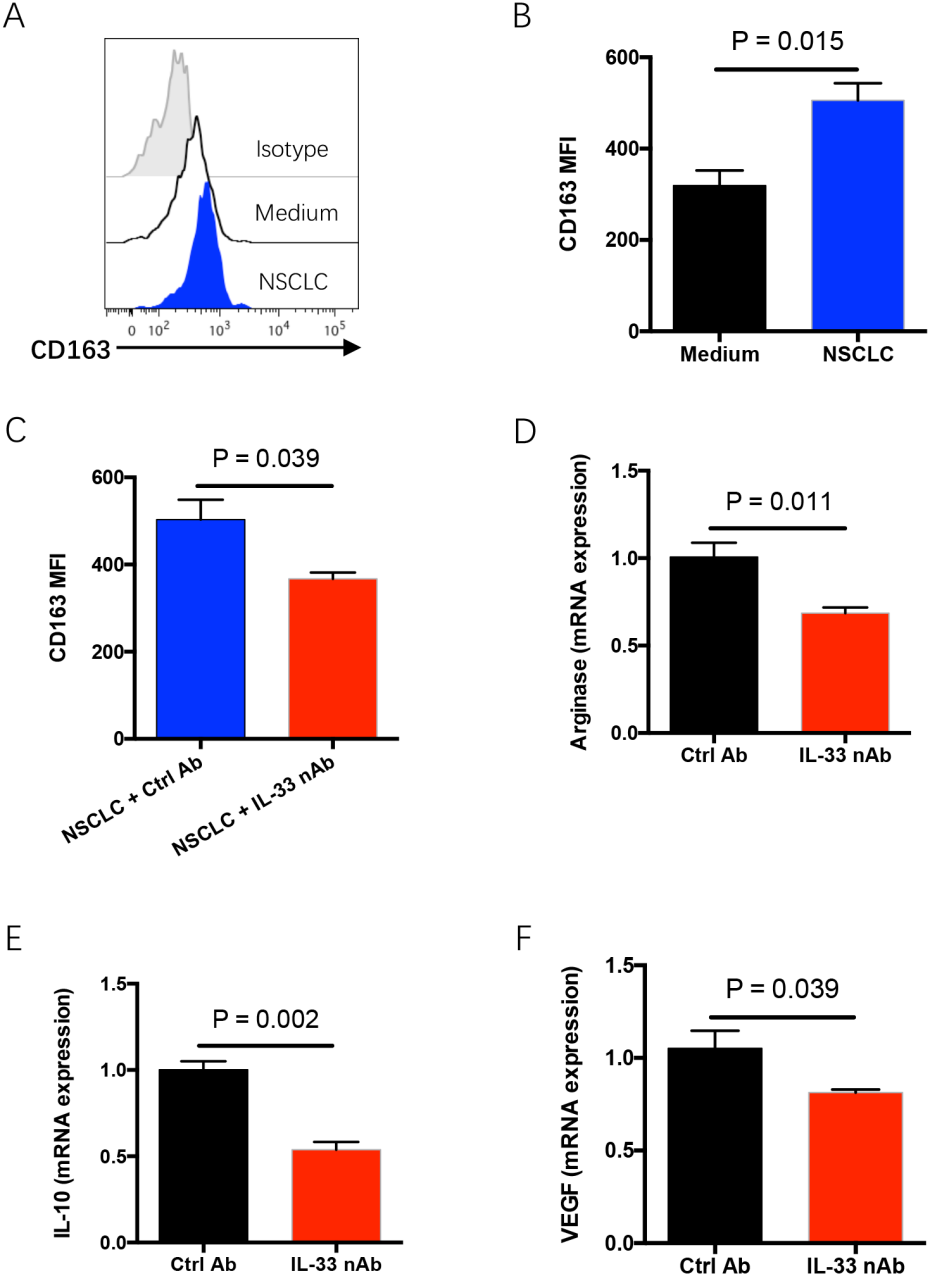
IL-33 blockade abrogates polarization of M2 TAMs in tumor microenvironment **(A, B)** Fresh TILs were treated with M-CSF (10 ng/ml) plus IL-10 (10 ng/ml) and co-cultured with or without NSCLC cells for 4 days. CD68-positive macrophages were gated from TILs and analyzed for CD163 expressions. Representative histograms and collective data (mean±SEM) from 6 independent experiments are shown. **(C)** Fresh TILs were incubated with M-CSF (10 ng/ml), IL-10 (10 ng/ml) and NSCLC cells in the presence or absence of IL-33 neutralizing antibody (10μg/ml) for 4 days. CD163 expression on CD68-positive macrophages was detected by flow cytometry. Shown are mean±SEM from 6 independent experiments. **(D-F)** Fresh tumor fragments from NSCLC patients were implanted into NSG mice, treated with IL-33 neutralizing antibody for 7 days and detected for expressions of M2 TAM-related genes including arginase 1 (D), IL-10 (E) and VEGF (F). Shown are mean±SEM from 6 independent experiments.

In NSCLC tumor tissues, IL-33 is robustly expressed in cancer cells while ST2 is expressed on both cancer cells and infiltrated lymphocytes [20], indicating IL-33 as a dual-function factor that could affect both NSCLC cells and immune surveillance in tumor microenvironments. Thus, freshly isolated tumor-infiltrating lymphocytes (TILs) and NSCLC cells were co-cultured to mimic the tumor microenvironment and analyzed for polarization of M2-polarized tumor-associated macrophages (TAMs), which is critical for tumor progression [23, 24]. Incubation with NSCLC cells enhanced the polarization of CD163-positive M2 TAMs in TILs, accompanied by an increased production of IL-10 (Figure 4A–4B and Supplemenatry Figure 1). The effect of NSCLC cells on polarization of M2 TAMs could be abrogated by IL-33 neutralization (Figure 4C and Supplemenatry Figure 1). To further confirm these findings *in vivo*, NSCLC tumor fragments were implanted into immune-deficient NSG mice and treated with IL-33 neutralizing antibody. IL-33 neutralization significantly inhibited the expressions of M2 TAM-related genes including arginase 1, IL-10 and VEGF in tumor tissues (Figure 4D–4F). These findings demonstrate that IL-33 blockade could inhibit the polarization of M2 TAMs, facilitating an anti-cancer tumor microenvironment.

### IL-33 blockade reduces accumulation of regulatory T cells in tumor microenvironment

**Figure 5:**
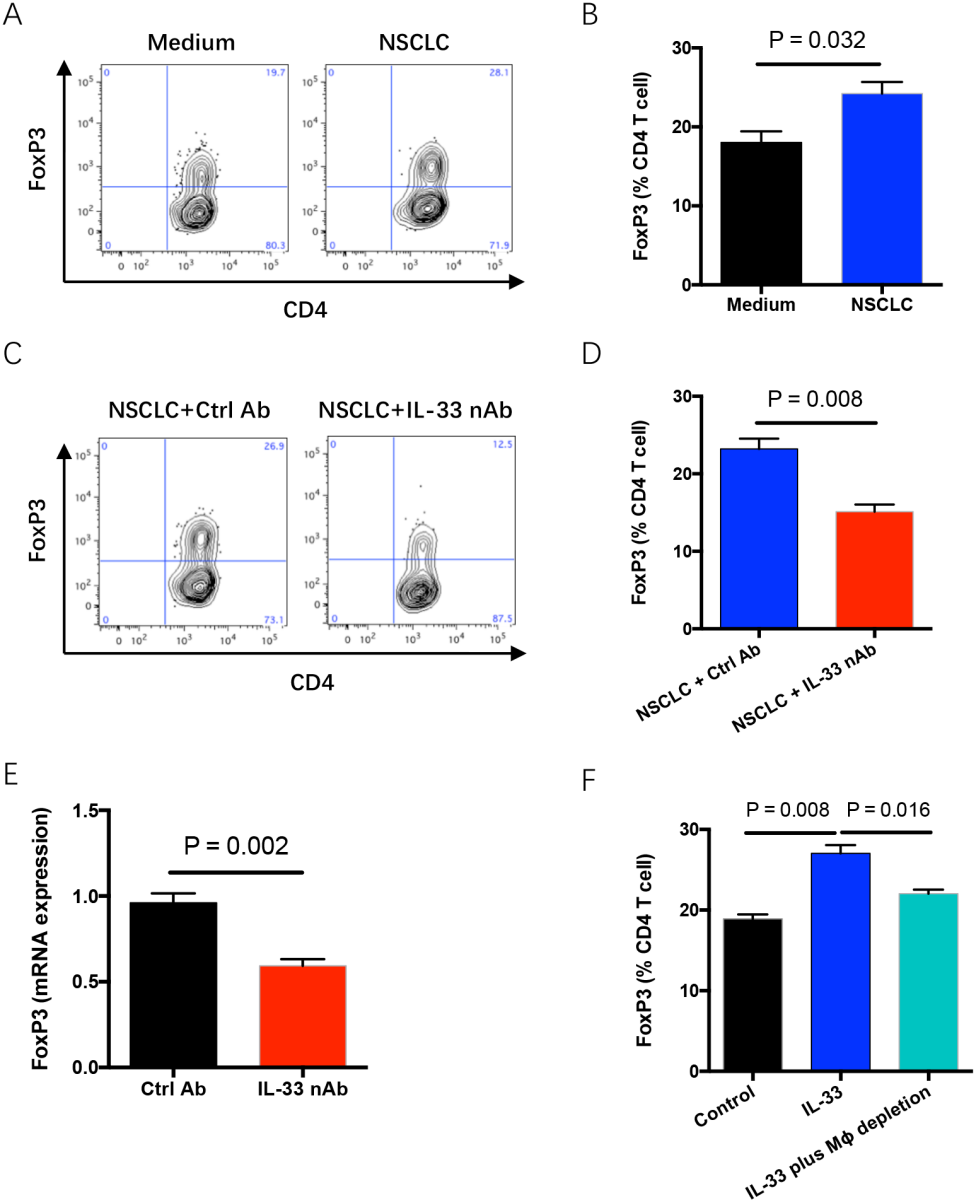
IL-33 blockade reduces Treg accumulation in tumor microenvironment **(A, B)** Fresh TILs were stimulated with anti-CD3 antibody (5μg/ml) plus anti-CD28 antibody (5μg/ml) and co-cultured with or without NSCLC cells for 4 days. Frequency of FoxP3-expressing CD4 T cells in TILs was analyzed by flow cytometry. Representatives and data (mean±SEM) from 5 independent experiments are shown. **(C, D)** Fresh TILs were incubated with anti-CD3 antibody (5μg/ml), anti-CD28 antibody (5μg/ml) plus NSCLC cells in the presence of IL-33 neutralizing antibody (10μg/ml) or the control for 4 days. Representatives and data (mean±SEM) from 5 independent experiments are shown. **(E)** Fresh tumor fragments from NSCLC patients were implanted into NSG mice, treated with IL-33 neutralizing antibody for 7 days and detected for FoxP3 expressions. Shown are mean±SEM from 6 independent experiments. **(F)** Fresh TILs with or without depletion of macrophages were stimulated with anti-CD3 plus anti-CD28 antibodies (5μg/ml) in the presence or absence of IL-33 protein (20 ng/ml) for 4 days. Shown are mean±SEM from 5 independent experiments.

Regulatory T cells (Tregs) are the suppressor of anti-cancer T effector cells and frequently enriched in tumor microenvironments [25, 26]. Co-culture with NSCLC cells upregulated the frequency of FoxP3-positive Treg cells in TILs, which could be blocked by IL-33 neutralization (Figure 5A–5D). *In vivo* confirmation of these data was performed in NSG mice with engraftment of human NSCLC fragments. IL-33 blockade dramatically reduced FoxP3 expression in NSCLC tumor tissues (Figure 5E). In consistent, IL-33 exerted a contributing effect on FoxP3 induction, increasing the frequency of Treg cells in TILs (Figure 5F). The phenomenon was partially dependent on M2 TAMs as it could be abrogated by depletion of macrophages (Figure 5F). It is reasonable because human M2 macrophages could robustly induce Treg differentiation and function [27]. Therefore, diminished Treg cells in tumor tissues by IL-33 blockade could contribute to limited NSCLC growth.

### IL-33 expression reflects tumor outgrowth and immune escape features in tumor tissues of NSCLC patients

**Figure 6:**
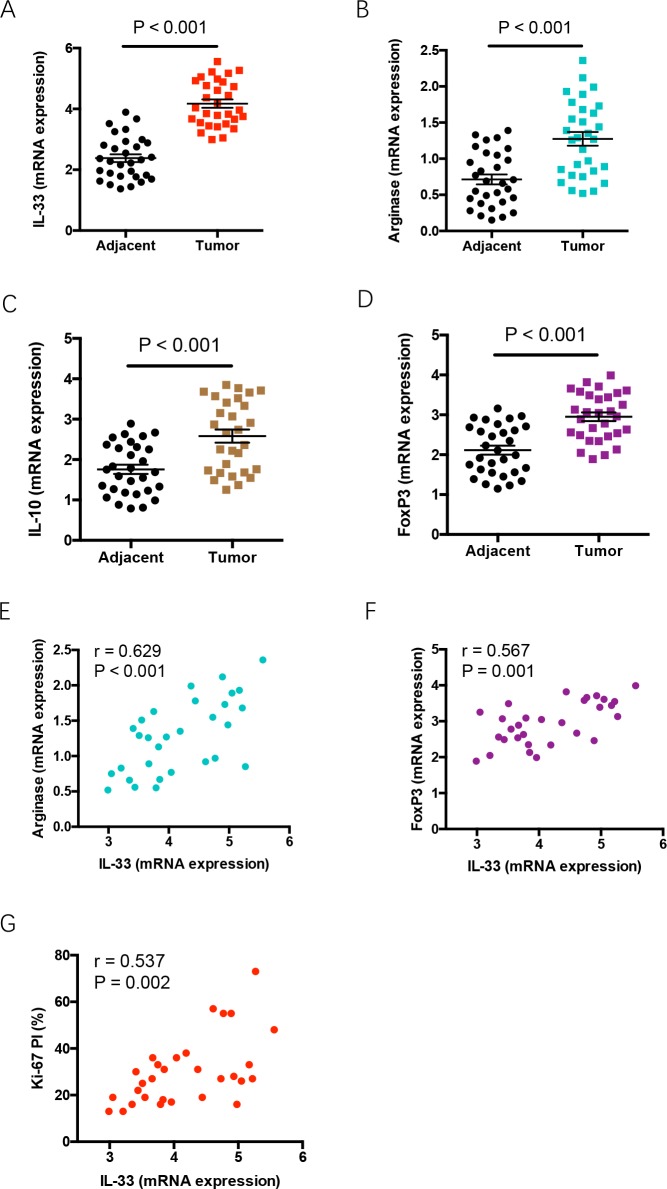
Increased IL-33 expression reflects tumor outgrowth and immune escape features in tumor tissues **(A-D)** Expressions of IL-33, arginase 1, IL-10 and FoxP3 were analyzed in tumor tissues and adjacent tissues from NSCLC patients (n=30) by qPCR. Shown are relative expression normalized to the internal control. **(E-G)** IL-33 expression in tumor tissues was analyzed for correlations with expressions of arginase 1 and FoxP3, and Ki-67 PI in NSCLC patients. Each dot represents the data from an individual patient.

To explore the *in vivo* relevance of above findings, we analyzed the correlation of IL-33 expression with Ki-67 proliferation index (PI), and expressions of arginase 1, IL-10 and FoxP3 in tumor tissues of NSCLC patients. We observed significantly higher expressions of IL-33, arginase 1, IL-10 and FoxP3 in NSCLC tumor tissues than adjacent tissues (Figure 6A–6D). Further, IL-33 expression levels were closely associated with expressions of arginase 1 and FoxP3 in NSCLC tissues (Figure 6E, 6F). IL-33 expression was positively correlated with Ki-67 PI of NSCLC patients (Figure 6G). These findings indicate a contributing function of IL-33 in promoting NSCLC proliferative survival, polarization of M2 TAMs and accumulation of Treg cells in NSCLC patients.

## DISCUSSION

In this study, we uncover IL-33 blockade as a novel and effective therapeutic for NSCLC patients using a preclinical model. Blocking IL-33 activity restricts tumor growth of NSCLC xenografts. Mechanistically, IL-33 blockade suppresses outgrowth of NSCLC cells, abrogates polarization of M2 TAMs and reduces accumulation of Treg cells, shaping immune surveillance in tumor microenvironments.

Immunotherapy is a new frontier in clinical treatment of NSCLC patients [28]. The therapeutic is based on the idea that immune surveillance is a crucial mechanism for host protection against carcinogenesis and thus improving the functional competence of immune effector cells would ultimately eliminate tumor cells [29]. Indeed, checkpoint immunotherapies with inhibitors targeting PD-L1/PD-1 have shown exciting results in clinical study [30]. But, cancer incidence increases ever year and cancer develops any way even in the host with a functioning immune system [31]. This opens a complex and dynamic process consisting of immune surveillance and tumor progression, termed as cancer immunoediting [31, 32]. Herein, we demonstrate that incubation with NSCLC cells facilitated polarization of M2 TAMs and accumulation of Treg cells in tumor microenvironments, suggesting cancer immunoediting as an efficient mechanism for immune escape of lung cancer cells.

IL-33 is expressed in high levels in cancer cells from NSCLC patients, supporting NSCLC outgrowth and metastasis [20]. Here we extend previous findings by providing evidence that IL-33 blockade efficiently limited NSCLC growth through direct and indirect pathways. IL-33 neutralization results in reduced proliferative survival of NSCLC cells, insufficient M2-polirzaied TAMs and diminished Treg cells in tumor tissues. In NSCLC patients, IL-33 expression levels were positively correlated with Ki-67 PI and expressions of M2 TAM- and Treg-related genes. In line with our findings, previous studies showed that IL-33 was able to facilitate polarization of M2 macrophages and promote the expansion and function of FoxP3-positive Treg cells [33–37]. Besides, we noticed that increased Treg cells by IL-33 was at least partly dependent on M2 TAMs. Depletion of macrophages significantly abrogated the effect of IL-33 on accumulation of Treg cells. Thus, we proposed an IL-33-M2 TAM-Treg pathway underlying progression of human lung cancer. IL-33 increased the frequency of Treg cells through both M2 TAM-dependent and -independent pathway. Combing these findings suggest IL-33 as a dual-function factor in NSCLC progression. IL-33 functions as an intrinsic molecular mechanism supporting NSCLC outgrowth and a tumor-derived factor involved in cancer immunoediting.

We would like to point out that there are some studies showed an anti-cancer activity of IL-33 using established cancer cell lines and murine models. Qin L et al showed that systemic administration of recombinant IL-33 dramatically inhibited the leukemia growth and prolonged the survival of leukemia-bearing mice by increasing the expansion and IFN-γ production of leukemia-reactive CD8 T cells [38]. Tumoral expression of IL-33 was also reported to inhibit tumor growth and modifies the tumor microenvironment through CD8 T cells, favoring tumor eradication [39, 40]. These data raised a possibility that IL-33 might exert anti-cancer activity under certain circumstances. Herein, we determined the pro-cancer activity of IL-33 in tumor progression using fresh isolated cancer cells from lung cancer patients. The distinct observations might due to different types of tumors or reflect a fact that human cancer cell lines could not fully represent the characteristics and heterogeneity of cancer cells from clinical patients.

Our current findings are obtained from a preclinical model with human NSCLC xenografts and thus have a close clinical relevance. IL-33 blockade could be a promising therapeutic strategy for NSCLC patients. However, we need to admit some limitations of this study. First, the sample size is relatively low and experiments on a large size of NSCLC samples would be valuable to substantiate these findings. Second, although the preclinical model with NSCLC xenografts in immune-deficient mice closely reflects an *in vivo* relevance and provides a useful platform to explore anti-cancer reagents, this model could not represent the complexity of NSCLC progression in clinical patients. In addition, precise molecular mechanisms connecting IL-33 and tumor outgrowth, as well as immune surveillance, in NSCLC patients are unclear. Therefore, there is still a long distance from current findings to clinical applications.

In essence, NSCLC-derived IL-33 supports tumor growth in an autocrine manner and educates immune surveillance in tumor microenvironments, favoring immune escape of tumor cells. IL-33 blockade restricts NSCLC outgrowth, abrogates polarization of M2 TAMs and reduces accumulations of Treg cells in tumor tissues, representing an effective and promising strategy for NSCLC treatment. These findings shed new insight into NSCLC pathogenesis and provide clues for developing novel therapeutics against NSCLC in clinical practice.

## MATERIALS AND METHODS

### Patients

Thirty-five patients with biopsy-proven diagnosis of NSCLC were enrolled in this study. Written informed consent was obtained from each individual patient before collecting surgical tissues and clinical parameters. Ki-67 proliferation index was evaluated by two pathologists in our institution. The clinical parameters of NSCLC patients were summarized in Supplementary Table 1. Experiments were performed in accordance with the 1964 Helsinki declaration including its later amendments and approved by Tongji Institutional Ethics Committee.

### Mice

Immune-deficient SCID mice, nude mice and NSG mice between 6 to 8 weeks of age were from Shanghai Laboratory Animal Center, CAS and Biocytogen LLC. Mice were housed under specific pathogen-free conditions and experiments were approved by Tongji Institutional Ethics Committee.

### Cells and reagents

NSCLC cells were isolated from surgical tissues with Clonogenic Tumor Cell Isolation Kit (Cell Biolabs) [20]. Digestion of NSCLC tissues was performed as previously described [41], and tumor infiltrating lymphocytes (TILs) were isolated with CD45 MicroBeads, human (Miltenyi Biotec). Depletion of macrophages from TILs was performed by using CD14 Microbeads, Human (Miltenyi Biotec). Cells were cultured in complete RPMI 1640 medium containing 10% heat-inactivated FBS (Gibco) supplemented with 2mM glutamine, 100IU/ml penicillin and 100 mg/ml streptomycin sulfate at 37°C under 5% CO2. Kits were used according to the manual's instructions.

Recombinant human IL-33 protein, human M-CSF protein, human IL-10 protein, human IL-33 neutralizing antibody and human ST2 neutralizing antibody were from R&D Systems. Anti-human CD3 antibody and anti-human CD28 antibody were from eBioscience. All reagents were used according to manufacturer's instructions.

### NSCLC xenografts and treatment

Establishment of NSCLC xenografts in immune-deficient mice were performed as previously described [21, 22, 42]. Fresh tumor samples from NSCLC patients were cut into pieces of 4-5mm and implanted subcutaneously into immune-deficient SCID mice. Tumor growth was measured with digital calipers by two researchers every week and tumors were removed into immune-deficient nude mice when the tumor size reached 500-600mm^3^. The mouse-to-mouse passage was performed to establish stable growth of NSCLC xenografts.

NSCLC xenografts in the fourth generation were used to evaluate therapeutic efficiency of IL-33 blockade. Mice bearing NSCLC xenografts were injected intraperitoneally with 1μg of human IL-33 protein, 100μg of IL-33 neutralizing antibody or 100μg of ST2 neutralizing antibody at three times a week for 4 weeks [43, 44]. Tumor size were measured with digital calipers by two researchers every week.

### MTT assay

NSCLC outgrowth capacity was detected by MTT assay [20]. NSCLC cells (6 × 10^3^ cells/well) were cultured with recombinant human IL-33 protein in the presence or absence of IL-33 neutralizing antibody and measured with MTT cell proliferation kit (Cayman Chemical) according to the manual's instructions.

### Transwell co-culture assay

Co-culture of TILs with NSCLC cells was performed with the trans-well assay [45]. Freshly isolated TILs (5 × 10^5^ cells) were placed in the lower compartment of Corning® Transwell® polyester membrane cell culture inserts. Same number of NSCLC cells were seeded in the upper compartment and co-cultured with TILs for 4 days. TILs were then collected for analyzing macrophages and Treg cells with flow cytometry.

### Flow cytometry

To analyze apoptosis of NSCLC cells, 2 × 10^5^ cells were stained for Annexin V with Annexin V-FITC Apoptosis Detection Kit (eBioscience) according to the manual's instructions. For detecting the frequencies of M2-TAMs and regulatory T cells, 2 × 10^5^ cells were treated with cell fix/perm buffer (BD Bioscience) and stained with FITC anti-human CD68 antibody, APC anti-human CD163 antibody, PE-Cy7 anti-human CD4 antibody, PE anti-human FoxP3 antibody or isotype controls (all from eBioscience) for 45 min at room temperature in the dark. Samples were analyzed on a LSR II flow cytometer (BD Biosciences) and data were analyzed with FlowJo software (Tree Star).

### Real-time PCR

Detection of mRNA expressions of human IL-33, arginase 1, VEGF and Foxp3 was performed using quantitative real-time PCR [20]. Total RNA was extracted using Ambion® Isolation Kit (Thermo Fisher). cDNA was synthesized with First-Strand cDNA Synthesis Kit (Thermo Fisher). qPCR was performed using SYBR Green Master Mixes (Thermo Fisher). Human IL-33, arginase 1, VEGFA, Foxp3 and GAPDH primers were obtained from Sino Biological Inc. GAPDH was used as an internal control.

### Statistical analyses

Data were presented as mean ± SEM. Mann-Whitney test and Spearman correlation were used for statistical analyses using the program PRISM 6.0 (GraphPad Software Inc). A value of P < 0.05 was considered statistically significant.

## SUPPLEMENTARY MATERIALS FIGURE AND TABLE



## Abbreviations

NSCLC: non-small-cell lung cancer
TAM: tumor-associated macrophage
Treg: regulatory T cell
