# Supplementary material for: IL-33 blockade suppresses tumor growth of human lung cancer through direct and indirect pathways in a preclinical model
Source: Oncotarget. 2017 Aug 2;8(40):68571–82. doi: 10.18632/oncotarget.19786 (PMC5620278; doi:10.18632/oncotarget.19786)
Supplement: Supplementary file 1 [file oncotarget-08-68571-s001.pdf]

IL-33 blockade suppresses tumor growth of human lung cancer through direct and indirect pathways in a preclinical model

SUPPLEMENTARY MATERIALS

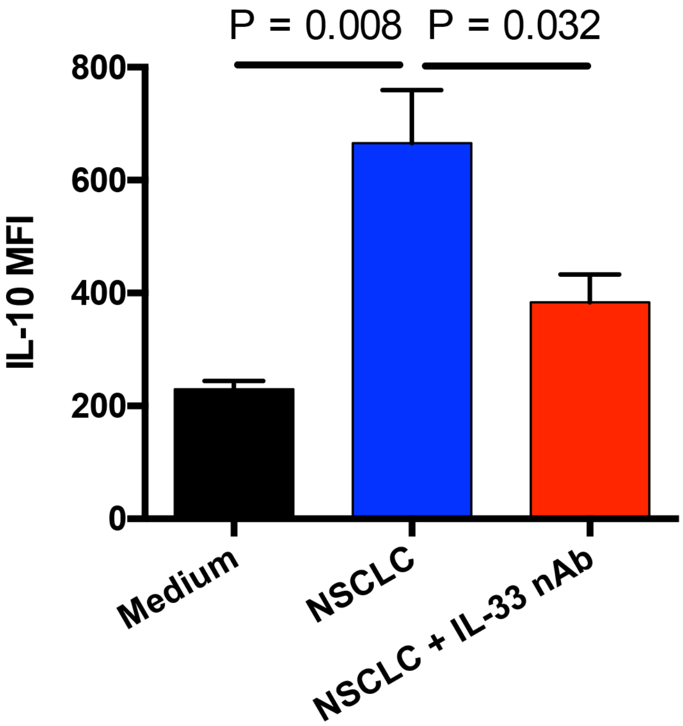

Supplemenaty Figure 1: NSCLC cells promotes IL-10 production in macrophages through IL-33 dependent manner. Fresh TILs were incubated with M-CSF (10ng/ml), IL-10 (10ng/ml) and NSCLC cells (1:1 ratio) in the presence or absence of IL-33 neutralizing antibody (10μg/ml) for 4 days. IL-10 expression in gated macrophages was detected by flow cytometry using PE-labeled human IL-10 antibody (eBioscience). Shown are MFI (mean±SEM) from 5 independent experiments.

Supplementary Table 1: Clinical characteristics of lung cancer patients

Clinical parameters	Number
Sex	
Male	22
Female	13
Age (years)	47-70
Median	61.3
Stages	
I	0
II	25
III	10
IV	0
Histological type	
Adenocarcinoma	23
Others	12

1. Clinical stage is according to TNM stage.
